# Hepatoprotective and Antioxidant Potential of Phenolics-Enriched Fraction of *Anogeissus acuminata* Leaf against Alcohol-Induced Hepatotoxicity in Rats

**DOI:** 10.3390/medsci10010017

**Published:** 2022-03-04

**Authors:** Lal Chand Pal, Shivankar Agrawal, Arti Gautam, Jayhind Kumar Chauhan, Chandana Venkateswara Rao

**Affiliations:** 1 Pharmacology Division, CSIR-National Botanical Research Institute, Lucknow 226001, Uttar Pradesh, India; lachandpal10@gmail.com (L.C.P.); aarti.goutam@gmail.com (A.G.); 2 Department of Phytochemistry, National Institute of Traditional Medicine, Indian Council of Medical Research (ICMR), Nehru Nagar, Belagavi 590010, Karnataka, India; 3 Department of Zoology, MahilaMahavidyalaya, Banaras Hindu University, Varanasi 221005, Uttar Pradesh, India; jayhindchauhan041@gmail.com

**Keywords:** hepatoprotective, alcohol-induced hepatotoxicity, antioxidant, interleukins

## Abstract

*Anogeissus acuminata* is used to treat wounds, diarrhoea, dysentery, and skin ailments. However, its hepatoprotective effect against ethanol-induced liver damage is yet to be reported. The phenolic-enriched ethyl acetate fraction of *Anogeissus acuminata* (AAE) was evaluated for hepatoprotective activity against ethanol-induced liver toxicity in rats. The intoxicated animals were treated with a phenolic-rich fraction of *Anogeissus acuminata* (AAE) (100 and 200 mg/kg) and silymarin (100 mg/kg). The antioxidant activity of AAE was analysed. Biochemical markers (ALT, AST, ALP, GGT, and TBL) for liver injury in ethanol-administered animals resulted in higher levels of key serum biochemical injury markers, as evidenced by increased levels of ALT (127.24 ± 3.95), AST (189.54 ± 7.56), ALP (263.88 ± 12.96), GGT (91.65 ± 3.96), and TBL (2.85 ± 0.12) compared to Group I ALT (38.67 ± 3.84), AST (64.45 ± 5.97), GGT (38.67 ± 3.84), and TBL (0.53 ± 064) (*p* < 0.05). AAE administration decreased serum biochemical liver injury markers as manifested in Group III animals’ ALT (79.56 ± 5.16), AST (151.76 ± 6.16), ALP (184.67 ± 10.12), GGT (68.24 ± 4.05), TBL (1.66 ± 0.082) (*p* < 0.05), and Group IV ALT (55.54 ± 4.35), AST (78.79 ± 4.88), ALP (81.96 ± 9.43), GGT (47.32 ± 2.95), TBL (0.74 ± 0.075) (*p* < 0.05). Group IV exhibited the most significant reduction in serum biochemical markers as compared to Group III (*p* < 0.05) and close to silymarin-treated Group V ALT (44.42 ± 3.15), AST (74.45 ± 5.75), ALP (67.32 ± 9.14), GGT (42.43 ± 2.54), TBL (0.634 ± 0.077). Gene expression indices and histoarchitecture were evaluated to demonstrate the potential of AAE. The bioactive fraction of *Anogeissus acuminata* was rich in phenolics and flavonoid content. GC–MS analysis identified gallic acid, palmitic acid, cis-10-heptadecenoic acid, 9-octadecenoic acid, epigallocatechin, 2,5-dihydroxyacetophenone, and catechin. Oral administration of AAE (100 and 200 mg/kg) lowered the elevated levels of the biochemical markers and interleukin, and enhanced the level of enzymatic antioxidant. It also downregulated the expression level of proapoptotic genes and upregulated the expression level of the antiapoptotic gene along with improved liver histopathology.

## 1. Introduction

Alcohol is the second most psychoactive or recreational substance consumed after caffeine. The long-term consumption of alcohol leads to an increased risk of major health problems such as injuries, violence, liver diseases, and cancer. According to the World Health Organisation, alcohol consumption is more harmful to hypercholesterolemia and hypertension than cigarette smoking is [1]. Alcohol causes oxidative stress in the liver cells, which leads to metabolic abnormalities such as the accumulation of acetaldehyde, damage to the cell membrane and mitochondria, hypoxia, a disrupted immune system, cytokine production, CYP2E1 activation, and iron mobilisation. The stages of alcoholic liver disease (ALD) are broadly classified as fatty liver/steatosis, alcoholic hepatitis, and liver cirrhosis. Intermediates produced during the reduction in oxygen may be responsible for the formation of ALD. A significant increase in the levels of free radicals is found (in human hepatocytes) immediately after receiving alcohol because ethanol or its metabolites may function as pro-oxidants or lead to a reduction in antioxidants in the body, and enhance the level of reactive oxygen species (ROS), which is the cause of chronic liver disease [2]. The liver is the organ responsible for the metabolism of ingested alcohol. Alcohol can be metabolised in the liver through both oxidative and nonoxidative mechanisms. Several enzymes are involved in these pathways, including the microsomal ethanol oxidising system (MEOS, CYP2E1) and catalase (less significant). The role of MEOS in ALD is limited to the production of acetaldehyde, which affects mitochondrial function. Excessive alcohol intake enhances the activity of cytochrome P450 2E1 (CYP2E1), which can generate acetaldehyde via the generation of reactive oxygen species (ROS). ROS in high concentrations can cause cellular dysfunction, and, in the worst circumstances, cell death. Both acetaldehyde and ROS can induce adverse consequences such as nausea, vomiting, itching, and a high pulse rate. To avoid serious liver damage, it is essential to eliminate excess ethanol and acetaldehyde [3]. ROS are extremely damaging agents that. cause significant damage to lipids, proteins, and DNA. Oxidative stress is caused by an imbalance between free radicals and antioxidants, which endangers human life by increasing the occurrence of cancer, diabetes, cardiac disease, and neurological problems [4]. As a result, there is a need to establish defensive mechanisms in order to survive and maintain a healthy lifestyle. Antioxidants are generally an effective approach to preventing oxidative stress by the mechanism of inhibiting ROS concentration by the breaking of chain reactions, free radical scavenging, and chelating transition metals that catalyse free radical formation [5]. Numerous synthetic antioxidants are commercially available to alleviate oxidative stress, but they have adverse side effects [6,7]. Plant-derived or herbal compounds may be effective as antioxidants because they do not have detrimental side effects because of their natural origin and composite character [8]. Plants are major sources of polyphenols. A class of compounds with a phenolic hydroxyl structure, they attract researcher attention for their potent antioxidant activities [9].

Many plants of the genus *Anogeissus* (Combretaceae) have been investigated for their biologically active components. Dhaura (Hindi) plant *Anogeissus acuminata* is widespread in the semideciduous forests of India, Bangladesh, Laos, Cambodia, Myanmar, Vietnam, Thailand, and Africa. In India, *A. acuminata* is traditionally used to treat wound healing, diarrhoea, dysentery, and skin problems [10]. This plant contains a significant level of antioxidants such as polyphenols, flavonoids, terpenoids, vitamin E, vitamin C, selenium, beta-carotene, and other carotenoids, which together play a key role in scavenging free radicals [8]. The present study was undertaken to estimate the hepatoprotective potential of the ethyl acetate fraction of *Anogeissus acuminata* leaf extract.

## 2. Materials and Methods

### 2.1. Chemicals and Kits

Ethylenediaminetetraacetic acid (EDTA) and Enhanced Avian HS RT-PCR kit were procured from Sigma Aldrich (St. Louis, MO, USA). The biochemical reagent kit was procured from Transasia Bio-Medicals Ltd. (Maharashtra, India). Ethanol (Sigma Aldrich, USA) and ELISA kit were procured from Elabscience. All other utilised chemicals and reagents were of analytical-grade, and procured from Merck and Hi-Media Pvt. Ltd. (Maharashtra, India).

### 2.2. Extract Preparation and Phytochemical Profiling

The leaf samples of *A. acuminata* were collected from the garden of the National Botanical Research Institute, Lucknow (India). Shade-dried samples were extracted with 70% *v/v* ethanol. Extract was employed in a rotary evaporator and freeze-dried in a lyophiliser at high vacuum and low temperature. The dried extract was further fractionated with solvent of different polarity indices (hexane, chloroform, and ethyl acetate). Among all fractions, the bioactive ethyl acetate (AAE) fraction was dried and stored for hepatoprotective investigation. Total phenolic content (TPC) was analysed by using the Ragazzi and Veronese (1973) method [11], and is expressed as mg gallic acid-equivalent (GAE)/gram AAE. Total flavonoid content (TFC) was investigated by the Oyaizu (1986) method [12] and expressed as mg quercetin-equivalent (QE)/gram AAE. The phytochemical characterisation of the bioactive fraction of *Anogeissus acuminata* leaf extract (AAE) was conducted via GC–MS. Sample (AAE) was derivatised by using *N*-methyl-*N*-(trimethylsilyl)trifluoroacetamide (MSTFA). The derivatised fraction was evaluated on the GC–MS instrument comprising a gas chromatograph (Thermo Trace GC Ultra) and mass spectrometers (Thermo Fisher DSQ II). Data were recorded by mass selective detector operating in the electron impact (EI) mode with 70 eV ionisation energy at an ionisation current of 2.0 mA and mass range of 50–800 m/z. The resultant chromatographic and mass data were acquired using Xcalibur software. The software depicts the investigation of the m/z ratio values of each metabolite fragments detected in mass spectra using GC-MS spectral library databases such as WILLY and NIST. The relative concentration of detected metabolites was calculated as a percent peak area [13].

### 2.3. In-Vitro Antioxidant Activity

Using the DPPH stable radical, the antioxidant potential of the bioactive fraction (AAE) was investigated [14]. The reducing potential of AAE was recorded at 515 nm with a calibration curve and determined by linear regression. Results obtained from AAE were compared with ascorbic acid, which is used as a standard antioxidant. DPPH radical inhibition was evaluated according to the equation.

DPPH˙ radical inhibition = Control − Sample/Control × 100

Ferric reducing power (RP) evaluation was estimated by the ferric reducing power assay and denoted as mg ascorbic acid equivalents (ASE) per gram AAE [15]. Total antioxidant capacity was determined by using the spectrophotometric method [16]. Ascorbic acid was used as the standard, and total antioxidant capacity is expressed as mg ascorbic acid equivalents (ASE) per mg AAE.

### 2.4. Experimental Animals

Male Sprague Dawley rats were used in this study according to the regulations of the Institutional Animal Care Committee, CPCSEA, India (reg. no. 1732/GO/Re/s/13/CPCSEA). Acute toxicity was analysed according to OECD Guideline 423 [17]. The administration of AAE at doses of 300 mg/kg b.w. was a safe dose that exhibited no abnormal behaviour or mortality in tested rats. Twenty-five rats were divided into five groups (each group had five animals). All animals were orally intoxicated with ethanol (7 g/kg) for 28 days except Group I animals. Groups III and IV received AAE 100 and 200 (mg/kg, p.o.) once daily, while Group V received silymarin 100 (mg/kg, p.o.) for up to two weeks. Group II received 0.5% sodium carboxyl methyl cellulose [18]. After a two-week treatment period, the animals were sacrificed on an overnight fast by cervical dislocation. Blood was collected and centrifuged at 1000× *g* for 15 min to isolate serum for the investigation of biochemical markers. Collected vital organs were washed with phosphate buffer saline (PBS), fixed in formalin for histological studies, and the remaining tissue was stored at −80 °C for enzymatic antioxidants and molecular analysis.

### 2.5. Determination of Biochemical Parameters

Biochemical parameters were analysed by using Biochemical kits (Transasia Bio-medicals Ltd.) from the collected blood serum (AST, ALT, ALP, GGT, and total bilirubin) with an autochemistry analyser (Csense 100).

### 2.6. Determination of Antioxidant Enzymes and Stress Markers

Liver tissue homogenisenate was obtained by the homogenisation of 400 mg hepatic tissue in phosphate buffer (10 mM, pH 7.4) containing KCl (1.15%) and EDTA (1.15%, pH 7.4) followed by centrifugation. Total protein content in the different samples was quantified by the method of Bradford (1976) [19] at 595 nm (Spectramax 340PC, Molecular Devices, San Jose, CA, USA) by using BSA as a standard protein, and expressed as mg/g FW. Superoxide dismutase (SOD) (EC 1.15.1.1) activity was evaluated by the method of Kakkar et al. (1984) [20]. The activity of catalase (CAT) (EC1.11.1.6) was estimated with the Aebi (1974) method [21] and exhibited as µmole H_2_O_2_ consumed/mg protein. Glutathione peroxidase (GPX) activity was investigated using the method of Rotruck (1973) [22]. Glutathione S-transferase (GST; EC 2.5.1.13) activity was evaluated by using the method of Habig (1974) [23]. The MDA test was conducted to analyse lipid peroxidation by the estimation of thiobarbituric acid reactive substances (TBARS) according to Hodges (1999) [24]. Reduced glutathione (GSH) content was determined by the method of Ellman (1961) [25].

### 2.7. Estimation of Interleukins and TNF-α in Hepatic Tissue

To estimate interleukins and TNF-α from liver tissue homogenate, an ELISA kit (Elabscience Biotech Co., Ltd., Wuhan, China) was used based on the principle of standard sandwich ELISA technology.

### 2.8. Gene Expression Analysis via Quantitative Real-Time PCR (qRT-PCR)

The TRizol reagent was used to isolate RNA. The NanoDropTm apparatus was used to measure the quality and concentration of RNA at 260/280 nm. Enhanced Avian HS RT-PCR kit was used to synthesise cDNA from isolated total RNA (Sigma-Aldrich, St. Louis, MO, USA). This cDNA was used as a template for qRT-PCR to assess total transcript levels in a StepOne real-time PCR system using SYBR Green PCR Master Mix (Applied Biosystems, Waltham, MA, USA). The 2^−ΔΔct^ method was used to examine the quantitative real-time expression of the genes [26]. Table S1 shows the primer sequences developed for each gene.

### 2.9. Statistical Analysis

All estimated results are the mean of five replicates. Data were examined with Duncan’s multiple-range test (DMRT) for the evaluation of the significant difference between means (*p* < 0.05). SD is depicted using the average of the five replicates.

## 3. Results

### 3.1. GC–MS Analysis of Ethyl Acetate Fraction of A. acuminata Identified Bioactive Phytochemicals

**Figure 1:**
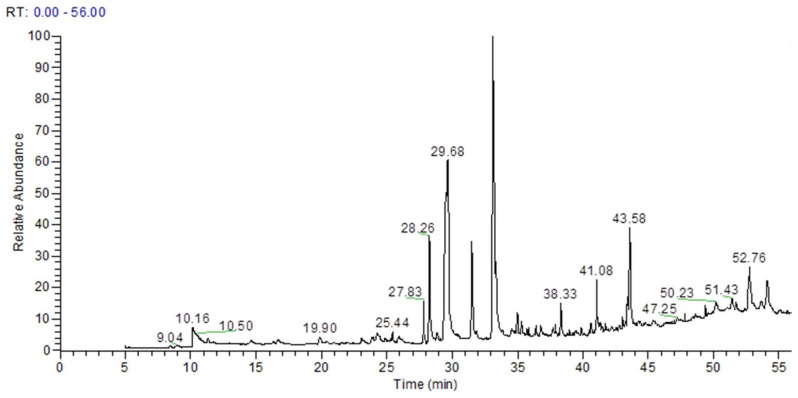
GC-MS chromatogram of ethyl acetate fraction of *Anogeissus acuminata* leaf extract.

The phytochemical profile of the ethyl acetate fraction of *A. aciminata* (AAE) was established out through mass spectrometry coupled with GC. In total, 25 compounds were identified in GC–MS analysis. The identified compounds and their retention time, molecular formula, and peak area (%) are given in Table 1. The major phytochemical compounds were gallic acid (4.66%), palmitic acid (19.78%), cis-10-heptadecenoic acid (5.38%), 9-Octadecenoic acid (22.39%), epigallocatechin (6.34%), 2,5-dihydroxyacetophenone (1.27%), and catechin (0.41%) (Figure 1). 

### 3.2. Total Phenolic and Flavonoid Content

The total phenolic and flavonoid content of plant extracts depends on the type of solvent used for their extraction procedure. The extraction yield of a 70% ethanolic extract of *A. acuminata* was 13.71% *w/w*. The total phenolic content of AAE was 313 ± 17.23 (mg GA eq./gram AAE), and the total flavonoid content was 124 ± 7.19 (mg RE eq./gram AAE). 

### 3.3. In Vitro Antioxidant Studies

DPPH is a free radical that can scavenge by accepting an electron from antioxidants and converting it into a stable diamagnetic molecule. The free radical scavenging effect of the active fraction on DPPH radicals increases with increasing concentration. At 5, 10, 15, 20, and 25 µg/mL, the scavenging activities of AAE on DPPH radical were 21%, 34%, 46%, 65% and 84%, respectively. The calculated IC_50_ was 16 µg/mL. The reducing power of AAE exhibits the capability of Fe^3+^ to Fe^2+^ reduction by antioxidant. The analysed reducing power of AAE was 341.21 ± 2.43 mg ascorbic acid equivalents/gram AAE. Total antioxidant capacity was 232 ± 2.89 mg ascorbic acid equivalent/gram of AAE.

### 3.4. Effect of AAE on Biochemical Parameters

**Figure 2:**
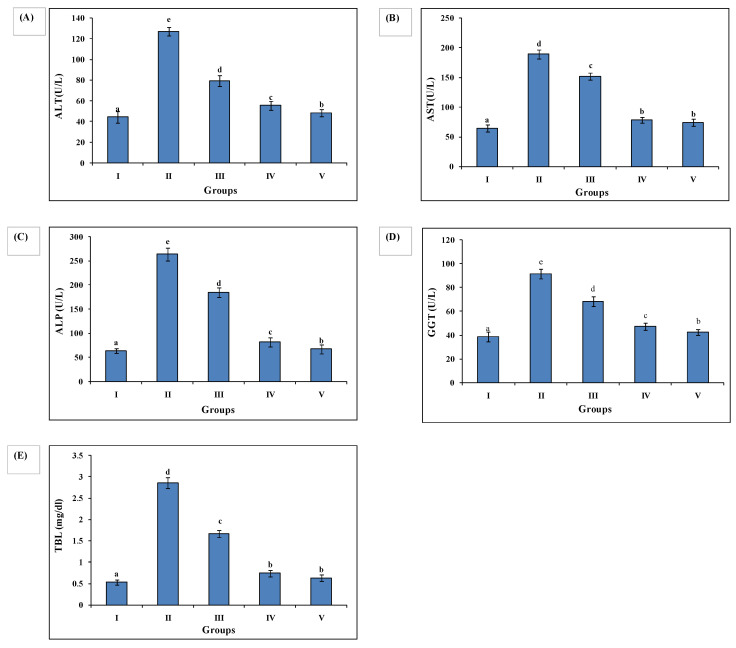
AAE maintained reduced levels of various biochemical parameters in Groups III and IV. (**A**) ALT (U/L), (**B**) AST (U/L), (**C**) GGT (U/L), (**D**) ALP (U/L), (**E**) TBL (mg/dL). All biochemical parameters were reversed by the test drug (AAE) administration in Groups III and Group IV, close to sylimarin-treated Group V, while Group II showed elevated levels. Duncan’s multiple-range test (DMRT) was used for the analysis of a significant difference between the means (*p* < 0.05) and compared each parameter. Bars with the same letters are not significantly different. All values are means of five replicates ± SD.

Ethanol administered to Group II animals resulted in higher levels of serum biochemical injury markers, as evidenced by increased levels of ALT (127.24 ± 3.95), AST (189.54 ± 7.56), ALP (263.88 ± 12.96), GGT (91.65 ± 3.96), and TBL (2.85 ± 0.12) compared to Group I ALT (38.67 ± 3.84), AST (64.45 ± 5.97), GGT (38.67 ± 3.84), and TBL (0.53 ± 064). AAE administration decreased serum biochemical markers as manifested in Group III animals’ ALT (79.56 ± 5.16), AST (151.76 ± 6.16), ALP (184.67 ± 10.12), GGT (68.24 ± 4.05), and TBL (1.66 ± 0.082), and Group IV ALT (55.54 ± 4.35), AST (78.79 ± 4.88), ALP (81.96 ± 9.43), GGT (47.32 ± 2.95), and TBL (0.74 ± 0.075). Group IV exhibited the most significant reduction in serum biochemical markers as compared to Group III, and close to silymarin-treated Group V ALT (44.42 ± 3.15), AST (74.45 ± 5.75), ALP (67.32 ± 9.14), GGT (42.43 ± 2.54), TBL (0.634 ± 0.077) (Figure 2).

### 3.5. AAE Treatment Improves Antioxidant Enzymes Activity and Lowers Lipid Peroxidation

**Figure 3:**
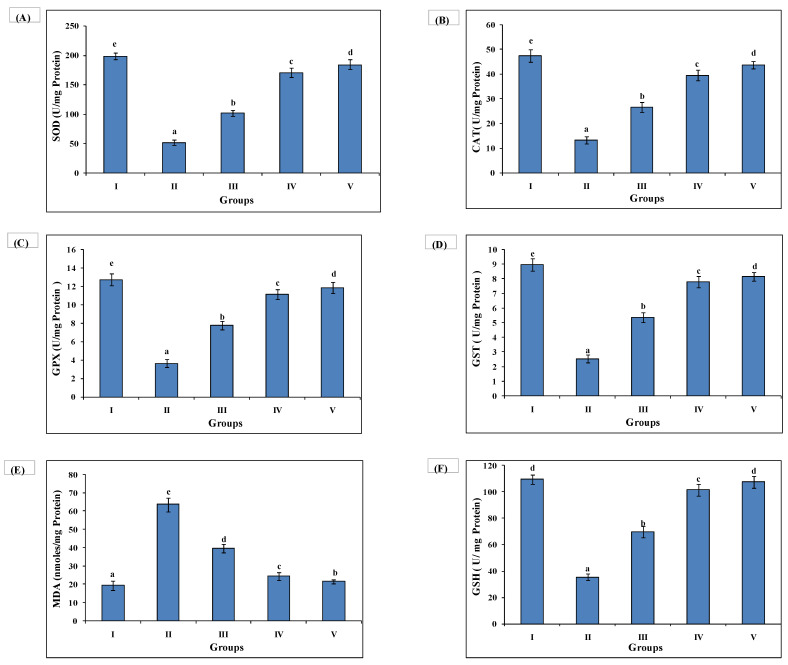
AAE treatment improves antioxidant enzyme activity and lowers lipid peroxidation. Higher antioxidant enzymes activities (**A**) SOD (U/mg protein), (**B**) catalase (U/mg protein), (**C**) GPX (U/mg protein), (**D**) GST (U/mg protein) (**E**) LPO (nmoles/mg protein) (**F**) GSH (U/mg protein) and lowered stress marker declined in AAE-treated Groups III and IV, where values of Group IV were close to those of silymarin-treated Group V. Group II had the lowest antioxidant activities and highest lipid peroxidation. Duncan’s multiple-range test (DMRT) was used for the analysis of a significant difference between the means (*p* < 0.05) and separately compared each parameter. Bars with the same letters are not significantly different. All values are means of five replicates ± SD.

Ethanol administration (7 g/kg) reduced antioxidant activity and concomitantly increased level of stress marker (MDA). Group II exhibited a decline in SOD (51.75 ± 4.19), catalase (13.25 ± 1.55), GPx (3.64 ± 0.41), GST (2.54 ± 0.26), GSH (35.55 ± 2.44) along with an elevated level of MDA (63.65 ± 3.76) as compared to untreated Group I SOD (198.53 ± 5.19), catalase (47.45 ± 2.55), GPx (12.72 ± 0.64), GST (8.96 ± 0.45), GSH (109.66 ± 3.56) and MDA (19.43 ± 2.44). AAE administration (100 and 200 mg/kg) enhanced the antioxidant activity and amelioration of MDA concentration in Group III SOD (101.87 ± 5.10), catalase (26.54 ± 1.95), GPx (7.76 ± 0.46), GST (5.37 ± 0.33), GSH (69.75 ± 4.01), MDA (39.74 ± 2.35) and, Group IV SOD (170.57 ± 8.31), catalase (39.54 ± 2.04), GPx (11.16 ± 0.52), GST (7.78 ± 0.38), GSH (101.43 ± 4.44), MDA (24.34 ± 2.12). Group IV exhibited the most significant antioxidant activity with decreased MDA level as in silymarin (100 mg/kg) administration Group V SOD (184.38 ± 8.20), catalase (43.67 ± 1.46), GPx (11.85 ± 0.59), GST (8.16 ± 0.30), GSH (107.42 ± 4.65), MDA (21.63 ± 1.12) (Figure 3).

### 3.6. ELISA Quantified Interleukins and TNF-α

**Figure 4:**
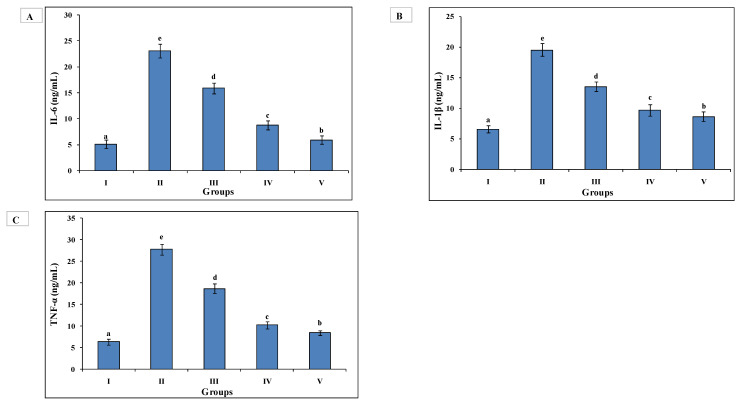
AAE treatment reduced levels of interleukins and TNF-α. AAE treatment reduced level of interleukins, (**A**) IL-6 (ng/mL), and (**B**) IL-1β (ng/mL) and, (**C**) TNF-α (pg/mL) in Groups III and IV. All values of Group IV close to silymarin-treated Group V. Group II had elevated levels of all parameters. Duncan’s multiple-range test (DMRT) was used for the analysis of a significant difference between the means (*p* < 0.05) and to separately compare each parameter. Bars with the same letters are not significantly different. All the values are means of five replicates ± SD.

Increased levels of IL-6 (23.121 ± 1.305), IL-1β (19.554 ± 1.032) and TNF-α (27.744 ± 1.247) were found in Group II animals as compared to Group I IL-6 (5.132 ± 0.861), IL-1β (6.567 ± 0.575) and TNF-α (6.343 ± 0.658). The increased levels of these markers were reversed back in the AAE-administered Group III, IL-6- (15.895 ± 1.068), IL-1β, (13.575 ± 0.787) and TNF-α (18.683 ± 1.150), and Group IV IL-6- (8.812 ± 0.847), IL-1β, (9.675 ± 0.896) and TNF-α (10.243 ± 0.866). Group IV exhibited significant restoration, as in Group V IL-6 (5.913 ± 0.795), IL-1β (8.667 ± 0.805) TNF-α (8.434 ± 0.561) (Figure 4).

### 3.7. Quantitative Real-Time Polymerase Chain Reaction (qRT-PCR) Investigation

**Figure 5:**
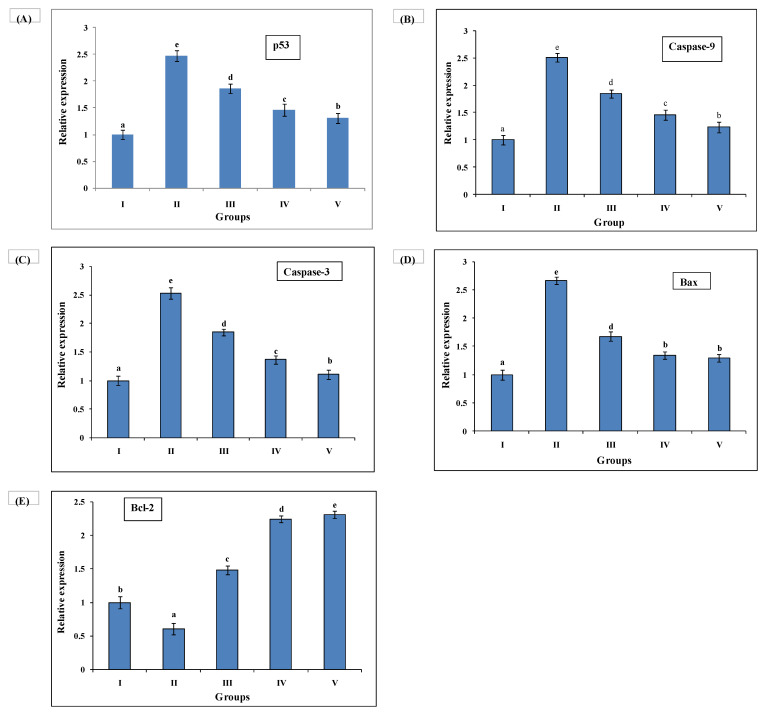
Relative expression of antiapoptotic and proapoptotic genes by qRT-PCR under MSG-induced liver toxicity. Relative expression of proapoptotic genes (**A**) p53, (**B**) caspase-9, (**C**) caspase-3, and (**D**) Bax was downregulated in all treatment groups as compared to Group II, and (**E**) expression of antiapoptotic gene Bcl-2 was upregulated. Duncan’s multiple-range test (DMRT) was used for analysis of a significant difference between means (*p* < 0.05) and separately compared each parameter. Bars with the same letters were not significantly different. All values are means of five replicates ± SD.

Ethanol administration promoted the expression of p53, Bax, caspase-3, and caspase-9, and significantly decreased the expression level of Bcl-2. AAE administration augmented the expression of p53, Bax, caspase-3, and caspase-9, and enhanced the expression level of Bcl-2 to protect ethanol intoxicated apoptosis and DNA damage in rat livers. The efficacy of AAE at 200 mg/kg was more significant than that at 100 mg/kg to improve altered gene expression, as in silymarin administration Group V. Gene-specific primers were used to investigate the relative expression of pro- and antiapoptotic genes. The GAPDH gene primer was taken as an endogenous control (Figure 5).

### 3.8. Histopathological Studies

**Figure 6:**
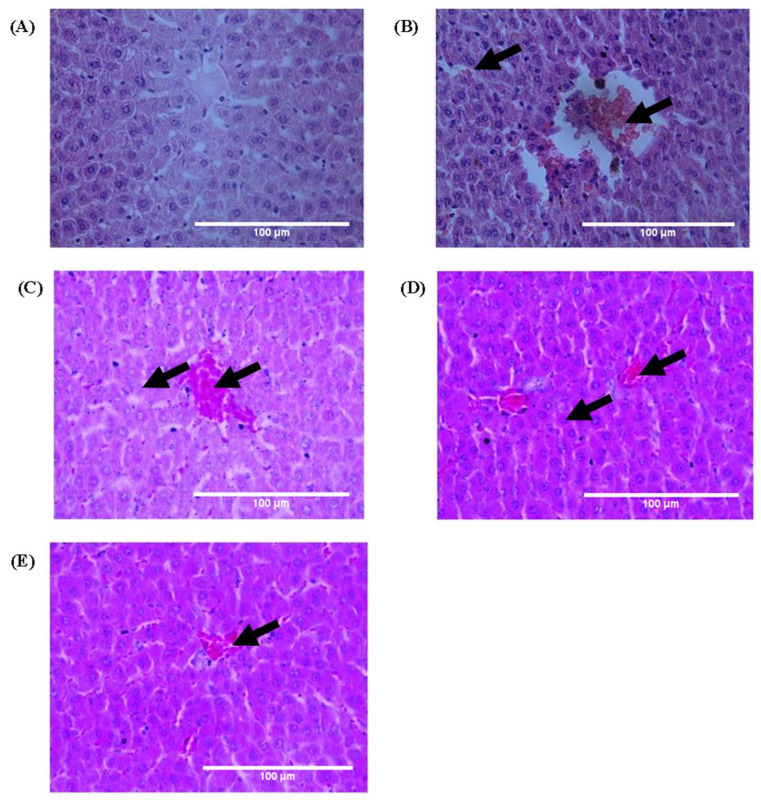
AAE improved architecture of hepatocyte lesions and inflammation. (**A**) H&E stain (40X) of Group I exhibited normal cells having well-preserved granulated cytoplasm and sinusoidal spaces with prominent nucleus and nucleolus. (**B**) Liver section from ethanol-intoxicated rats exhibited disarrangement of normal hepatic cells with centrilobular necrosis, vascular and cellular degeneration, with inflammation. (**C**–**E**) Administration of AAE in Groups III and IV exhibited reduction in necrosis with decreased inflammation, and improved the architecture of hepatocytes; improvement level in Group IV was much better than that of Group III and close to that of Group V. Arrows indicates the improvement in centrilobular necrosis, vascular, cellular degeneration and inflammation after AEE treated animals.

Histopathological examination of the liver section was observed with a microscope at 40X. H&E-stained liver section of Group I animals showed normal cell morphology with clear cellular boundaries, nucleus, and nucleolus with well-preserved granulated cytoplasm. No steatosis or inflammatory cells were observed compared to ethanol-intoxicated Group II animals’ liver sections. Ethanol-intoxicated animals in Group II exhibited a disarrangement of hepatocytes and number of inflammatory cells with cellular degeneration having centrilobular necrosis (Figure 6B). AAE-administered Groups III and IV showed improved hepatocyte architecture, reduction in inflammatory cells, and necrosis with clear nucleus and nucleolus, as in silymarin-treated Group V (Figure 6).

## 4. Discussion

The liver is the essential vital organ that metabolises various toxins and nutrients to keep the healthy human body in normal biochemical and physiological action; it should always function properly. GC–MS analysis of *A. acuminanata* revealed valuable pharmacologically active phytocompounds. The identified compounds were gallic acid (4.66%), palmitic acid (19.78%), cis-10-heptadecenoic acid (5.38%), 9-octadecenoic acid (22.39%), epigallocatechin (6.34%), 2,5-dihydroxyacetophenone (1.27%), and catechin (0.41%) (Table 1). Gallic acid and epigallocatechin have hepatoprotective properties in rat livers, as evidenced by a significant reduction in liver injury markers with CYP2E1, and increased antioxidant enzyme activity [2,27], indicating a strong therapeutic potential of gallic acid in this experimental liver disorder due to its potent antioxidant properties, DPPH scavenging activity, and reducing power assay. Palmitic acid and its derivatives are hepatoprotective and have anticancer activity [9]. 9-Octadecenoic acid showed antiandrogenic, 5-alpha reductase-inhibiting, cancer-preventive, anti-inflammatory, anaemiagenic, insectifuge, dermatitigenic, and hypocholesterolemic activity [28]. Thus, these pharmacologically active phytocompounds may exhibit potent restorations of altered biochemical injury markers in ethanol-intoxicated animals. The hepatoprotective potential of AAE was due to its valuable antioxidant properties, or phytocompound like phenolics and flavonoids, which reduced oxidative stress and its complications, such as ethanol-induced hepatic damage and inflammation [29,30,31,32]. DPPH (DPPH•) is a persistent radical that can accept an electron or hydrogen radical to produce a stable diamagnetic compound, leading to a change in colour. The percentage of DPPH colour change is widely used to estimate radical scavenging efficiency [33,34]. The existence of reducing agents (i.e., antioxidants) enables the Fe^3+^/ferricyanide complex to be reduced to ferrous form. As a result, evaluating the production of Perl’s Prussian blue at 700 nm can measure Fe^2+^ concentration. AAE’s reducing ability was enhanced with concentrations [35]. Ethanol is a unique substance with a rapid action owing to its solubility in both water and lipids, which is absorbed from the stomach and intestine and then rapidly diffuses into the blood circulation, where it is dispersed throughout the body. Hepatocellular necrosis is caused by alcohol use, resulting in an increase in serum marker enzymes released into the bloodstream [2]. Elevated ALT, AST, ALP, GGT, and TBL levels are prominent biomarkers of hepatic damage [9]. It also increases the production of reactive oxygen species inside the living system, since ethanol is extensively metabolised by the microsomal oxidising system to acetaldehyde and ultimately to acetate through cytochrome P450 [31]. Steatosis is the most significant alteration that happens in the liver following alcohol use. As a result of redox state imbalance, lipid peroxidation occurs [36]. In organisms, GSH is an essential antioxidant capable of reducing damage produced by reactive oxygen species. MDA is produced by lipid peroxidation and is a sign of oxidative stress. Free radicals and oxidative stress caused by ethanol promote MDA overproduction and GSH depletion [37]. Excessive free radicals are likely to elicit Kupffer cells, which can regulate the inflammatory process in the liver by releasing TNF-alpha and other pro-inflammatory cytokines [38]. Increased proinflammatory mediators and cytokines (e.g., TNF-α, IL-1 β, and IL-6) aggravated in ethanol-induced groups may have been through NF-κB activation [39]. The potent effect of AAE reduced free radicals and apoptotic properties by the enhanced antioxidant capacity against oxidative stress induced by alcohol for hepatoprotection. Histopathological examination revealed the hepatoprotective properties of AAE. AAE at a dosage of 200 mg/kg reflected more effective results, as in silymarin at 100 mg/kg. In the current study, we examined the gene expression levels of Bax, Bcl2, caspases3, caspases 9, and Bcl2 to evaluate apoptosis. The Bcl-2 family is associated with the initiation of the mitochondrial apoptotic pathway [40]. Bax, a proapoptotic protein, translocates to mitochondria and forms complexes with Bcl2. Increasing the Bax/Bcl2 ratio induces mitochondrial defects, resulting in the release of cytochrome C [41]. Caspases are proteolytic enzymes that cleave specific proteins in the nucleus and cytoplasm to damage the cell. When mitochondrial cytochrome c is released into the cytoplasm and activates caspase 9, which eventually activates caspase 3 via intrinsic apoptotic pathways. Cell membrane death receptors can activate initiator caspases, which leads to caspase-3 activation via extrinsic apoptosis pathways [4]. On the basis of real-time PCR, increased mRNA levels of the Bax/Bcl2 ration, and levels of caspases 3 and 9 were observed in the livers of ethanol-intoxicated rats, indicating that ethanol might reduce the level of Bcl2 while improving the level of Bax, which was consistent with our findings [42]. AAE might decrease mRNA expression levels of the Bax/Bcl2 ratio in the livers of intoxicated rats. As a result, AAE was highly hepatoprotective against ethanol toxicity, as was silymarin. All of the mentioned positive attributes confirmed the protective effects of AAE on ethanol toxicity symptoms via antilipid peroxidation, and antiapoptotic and antioxidant capabilities in our present study. The phenolics-enriched fraction of *Anogeissus acuminata* plays a significant role in hepatoprotective functions. As a result, in future studies, the potent phytocompounds will be isolated from the bioactive fraction, and their activities and mechanisms related to apoptosis will be explored.

## 5. Conclusions

According to the findings of this study, *Anogeissus acuminata* (AAE) may be useful in the treatment of alcohol-induced hepatotoxicity and oxidative stress. AAE showed significant hepatoprotective activity according to the results of various biomarkers. These findings contribute to the significance of the bioactive fraction’s constituents in hepatoprotective activity. Antioxidant, anti-inflammatory, and antiapoptotic properties of AAE may justify its beneficial impacts on alcoholic liver injury. Before AAE can be used in humans, extensive clinical studies must be carried out to demonstrate its safety and effectiveness. This study demonstrates the plant’s hepatoprotective properties, which could lead to the development of new hepatoprotective herbal medicines to treat alcohol-induced liver injury.

## Figures and Tables

**Table 1:** Phytocompounds identified in bioactive fraction of *A. acuminata* leaf extract.

RT(min)	Metabolites	Formula	Area	%Area
10.16	1H-Imidazole	C_3_H_4_N_2_	4.27 × 10^8^	2.74
14.56	Butanedioic acid, 2TMS	C_10_H_22_O_4_Si_2_	83,274,987	0.53
16.66	Tartaric acid (2R,3R)-, 3TMS	C_13_H_30_O_6_Si_3_	91,428,233	0.59
24.87	Shikimic acid, 4TMS derivative	C_19_H_42_O_5_Si_4_	23,304,997	0.15
25.44	Methyl galactoside (1S,2R,3S,4S,5R)-, 4TMS	C_19_H_46_O_6_Si_4_	82,226,063	0.53
27.83	2,5-Dihydroxyacetophenone, 2TMS derivative	C_14_H_24_O_3_Si_2_	1.98 × 10^8^	1.27
28.26	Gallic acid, 4TMS derivative	C_19_H_38_O_5_Si_4_	7.27 × 10^8^	4.66
28.87	Gibberellic acid, methyl ester	C_20_H_24_O_6_	79,478,987	0.51
29.68	Palmitic Acid, TMS derivative	C_19_H_40_O_2_Si	3.09 × 10^9^	19.78
31.52	cis-10-Heptadecenoic acid, trimethylsilyl ester	C_20_H_40_O_2_Si	8.39 × 10^8^	5.38
33.11	9-Octadecenoic acid, (E)-, TMS derivative	C_21_H_42_O_2_Si	3.49 × 10^9^	22.39
34.98	10-Nonadecenoic acid, (Z)-, TMS derivative	C_22_H_44_O_2_Si	1.42 × 10^8^	0.91
35.29	Linoleic acid trimethylsilyl ester	C_21_H_40_O_2_Si	1.15 × 10^8^	0.74
36.41	Arachidic acid, TMS derivative	C_23_H_48_O_2_Si	92,205,575	0.59
38.33	1-Monopalmitin, 2TMS derivative	C_25_H_54_O_4_Si_2_	2.02 × 10^8^	1.29
40.65	2-Palmitoylglycerol, 2TMS derivative	C_25_H_54_O_4_Si_2_	1.14 × 10^8^	0.73
41.08	1-Monooleoylglycerol, 2TMS derivative	C_27_H_56_O_4_Si_2_	4.39 × 10^8^	2.81
43.07	13-Docosenoic acid, (Z)-, TMS derivative	C_25_H_50_O_2_Si	59,210,641	0.38
43.58	Epigallocatechin (6TMS)	C_33_H_62_O_7_Si_6_	9.89 × 10^8^	6.34
44.24	Catechine (2R-E)-, 5TMS derivative	C_30_H_54_O_6_Si_5_	63,753,701	0.41
47.8	β-Sitosterol, TMS derivative	C_32_H_58_OSi	32,528,905	0.21
48.66	(+)-Prostaglandin F2α, 4TMS derivative	C_20_H_34_O_5_ · C_4_H_11_NO_3_	1.59 × 10^8^	1.02
51.43	1,3-Dipalmitin, TMS derivative	C_38_H_76_O_5_Si	1.42 × 10^8^	0.91
52.76	1,2-Dipalmitin, TMS derivative	C_38_H_76_O_5_Si	6.17 × 10^8^	3.91
54.12	1,3-Dipalmitin, TMS derivative	C_38_H_76_O_5_Si	4.51 × 10^8^	2.89

## Data Availability

Not applicable.

## Supplementary Materials

The following are available online at https://www.mdpi.com/article/10.3390/medsci10010017/s1. Table S1: sequences of primers used in PCR reactions.

## Abbreviations

TPC, total phenolic contents; TFC, total flavonoid contents DPPH, 2,2-diphenyl-1 picrylhydrazyl radical; FRAP, ferric reducing antioxidant power; T-AOC, total antioxidant capacity; BW, body weight; CAT, catalase; GPX, glutathione peroxidase; SOD, superoxide dismutase; MDA, malondialdehyde; GAE, gallic acid equivalents; ROS, reactive oxygen species; RT-PCR, reverse transcriptase polymerase chain reaction.

## References

[1] Nagappan A., Kim J.-H., Jung D.Y., Jung M.H. (2020). Cryptotanshinone from the Salvia miltiorrhiza bunge attenuates ethanol-induced liver injury by activation of AMPK/SIRT1 and Nrf2 signaling pathways. Int. J. Mol. Sci..

[2] Shukla I., Azmi L., Gupta S.S., Upreti D.K., Rao C.V. (2019). Amelioration of anti-hepatotoxic effect by Lichen rangiferinus against alcohol induced liver damage in rats. J. Ayurveda Integr. Med..

[3] Akter R., Kwak G.Y., Ahn J.C., Mathiyalagan R., Ramadhania Z.M., Yang D.C., Kang S.C. (2022). Protective Effect and Potential Antioxidant Role of Kakadu Plum Extracts on Alcohol-Induced Oxidative Damage in HepG2 Cells. Appl. Sci..

[4] Pal L.C., Gautam A., Pande V., Rao C. (2021). Anticancer property of *Selaginella bryopteris* (L.) Bak. against hepatocellular carcinoma in vitro and in vivo. Phytomedicine Plus.

[5] Sharma U.K., Kumar R., Gupta A., Ganguly R., Singh A.K., Ojha A.K., Pandey A.K. (2019). Ameliorating efficacy of eugenol against metanil yellow induced toxicity in albino Wistar rats. Food Chem. Toxicol..

[6] Silva M.M., Lidon F.C. (2016). An overview on applications and side effects of antioxidant food additives. Emir. J. Food Agric..

[7] Sarangarajan R., Meera S., Rukkumani R., Sankar P., Anuradha G. (2017). Antioxidants: Friend or foe?. Asian Pac. J. Trop. Med..

[8] Mendoza-Wilson A.M., Castro-Arredondo S.I., Espinosa-Plascencia A., del Refugio Robles-Burgueño M., Balandrán-Quintana R.R., del Carmen Bermúdez-Almada M. (2016). Chemical composition and antioxidant-prooxidant potential of a polyphenolic extract and a proanthocyanidin-rich fraction of apple skin. Heliyon.

[9] Pal L.C., Prateeksha, Singh B.N., Pande V., Rao C.V. (2021). Phenolics-Enriched Fraction of Pterospermum Lanceifolium Roxb. efficiently Reverses the Hepatocellular Carcinoma in NDEA-Induced HCC Rats. Nutr. Cancer.

[10] Singh D., Baghel U.S., Gautam A., Baghel D.S., Yadav D., Malik J., Yadav R. (2016). The genus Anogeissus: A review on ethnopharmacology, phytochemistry and pharmacology. J. Ethnopharmacol..

[11] Ragazzi E., Veronese G. (1973). Quantitative analysis of phenolic compounds after thin-layer chromatographic separation. J. Chromatogr. A.

[12] Oyaizu M. (1986). Studies on products of browning reaction antioxidative activities of products of browning reaction prepared from glucosamine. Jpn. J. Nutr. Diet..

[13] Bhatia A., Bharti S.K., Tripathi T., Mishra A., Sidhu O.P., Roy R., Nautiyal C.S. (2015). Metabolic profiling of Commiphora wightii (guggul) reveals a potential source for pharmaceuticals and nutraceuticals. Phytochemistry.

[14] Yen G.C., Duh P.D. (1994). Scavenging effect of methanolic extracts of peanut hulls on free-radical and active-oxygen species. J. Agric. Food Chem..

[15] Apati P., Szentmihalyi K., Kristo S.T., Papp I., Vinkler P., Szoke E., Kery A. (2003). Herbal remedies of Solidago—correlation of phytochemical characteristics and antioxidative properties. J. Pharm. Biomed. Anal..

[16] Prieto P., Pineda M., Aguilar M. (1999). Spectrophotometric Quantitation of Antioxidant Capacity through the Formation of a Phosphomolybdenum Complex: Specific Application to the Determination of Vitamin E. Anal. Biochem..

[17] OECD (1994). OECD Guidelines for the Testing of Chemical.

[18] Sapakal V., Shikalgar T., Ghadge R., Adnaik R., Naikwade N., Magdum C. (2008). In vivo screening of antioxidant profile: A review. J. Herb. Med. Toxicol..

[19] Bradford M.M. (1976). A rapid and sensitive method for the quantitation of microgram quantities of protein utilizing the principle of protein-dye binding. Anal. Biochem..

[20] Kakkar P., Das B., Viswanathan P. (1984). A modified spectrophotometric assay of superoxide dismutase. Indian J. Biochem. Biophys..

[21] Aebi H. (1974). Catalase. Methods of Enzymatic Analysis.

[22] Rotruck J., Pope A., Ganther H.E., Swanson A., Hafeman D.G., Hoekstra W. (1973). Selenium: Biochemical role as a component of glutathione peroxidase. Science.

[23] Habig W.H., Pabst M.J., Jakoby W.B. (1974). Glutathione S-transferases: The first enzymatic step in mercapturic acid formation. J. Biol. Chem..

[24] Hodges D.M., DeLong J.M., Forney C.F., Prange R.K. (1999). Improving the thiobarbituric acid-reactive-substances assay for estimating lipid peroxidation in plant tissues containing anthocyanin and other interfering compounds. Planta.

[25] Ellman G.L., Courtney K.D., Andres Jr V., Featherstone R.M. (1961). A new and rapid colorimetric determination of acetylcholinesterase activity. Biochem. Pharmacol..

[26] Livak K.J., Schmittgen T.D. (2001). Analysis of relative gene expression data using real-time quantitative PCR and the 2−ΔΔCT method. Methods.

[27] Baliga M.S., Shivashankara A.R., Venkatesh S., Bhat H.P., Palatty P.L., Bhandari G., Rao S. (2019). Phytochemicals in the prevention of ethanol-induced hepatotoxicity: A revisit. Diet. Interv. Liver Dis..

[28] Jananie R., Priya V., Vijayalakshmi K. (2011). Determination of bioactive components of Cynodon dactylon by GC-MS analysis. NY Sci. J..

[29] Chetia B., Gogoi S. (2011). Antibacterial activity of the methanolic extract of stem bark of Spondias pinnata, Moringa oleifera and Alstonia scholaris. Asian J. Tradit. Med..

[30] Arif M., Sheeba Fareed M., Rahman A. (2016). Stress relaxant and antioxidant activities of acid glycoside from Spondias mangifera fruit against physically and chemically challenged albino mice. J. Pharm. Bioallied Sci..

[31] Hoskeri H.J., Krishna V., Kumar B.V., Shridar A., Babu K.R., Sudarshana M. (2012). In vivo prophylactic effects of oleanolic acid isolated from chloroform extract of Flaveria trinervia against ethanol induced liver toxicity in rats. Arch. Pharmacal. Res..

[32] Chaturvedi S., Drabu S., Sharma M. (2012). Anti-inflammatory and analgesic activity of *Tamarix gallica*. Int. J. Pharm. Sci.

[33] Özdemir Ö. (2020). Bis-azo-linkage Schiff bases—Part (II): Synthesis, characterization, photoluminescence and DPPH radical scavenging properties of their novel luminescent mononuclear Zn (II) complexes. J. Photochem. Photobiol. A Chem..

[34] Agrawal S., Barrow C.J., Adholeya A., Deshmukh S.K. (2021). Unveiling the dermatological potential of marine fungal species components: Antioxidant and inhibitory capacities over tyrosinase. Biotechnol. Appl. Biochem..

[35] Kamath B.R., Kizhedath S. (2018). In vitro study on antioxidant activity of methanolic leaf extract of *Cassia fistula* Linn. Int. J. Basic Clin. Pharm..

[36] Lee J., Yang J., Jeon J., Jeong H.S., Lee J., Sung J. (2018). Hepatoprotective effect of esculetin on ethanol-induced liver injury in human HepG2 cells and C57BL/6J mice. J. Funct. Foods.

[37] Carrero R.J., Husain K. (2005). Renal oxidative stress in chronic alcohol-induced hypertension. Ethn. Dis..

[38] Jiang W., Gao M., Sun S., Bi A., Xin Y., Han X., Wang L., Yin Z., Luo L. (2012). Protective effect of L-theanine on carbon tetrachloride-induced acute liver injury in mice. Biochem. Biophys. Res. Commun..

[39] Tak P., Rigby W., Rubbert-Roth A., Peterfy C., Van Vollenhoven R., Stohl W., Hessey E., Chen A., Tyrrell H., Shaw T. (2011). Inhibition of joint damage and improved clinical outcomes with rituximab plus methotrexate in early active rheumatoid arthritis: The IMAGE trial. Ann. Rheum. Dis..

[40] Lee T.Y., Chang H.H., Wang G.J., Chiu J.H., Yang Y.Y., Lin H.C. (2006). Water-soluble extract of Salvia miltiorrhiza ameliorates carbon tetrachloride-mediated hepatic apoptosis in rats. J. Pharm. Pharmacol..

[41] Naseri M.H., Mahdavi M., Davoodi J., Tackallou S.H., Goudarzvand M., Neishabouri S.H. (2015). Up regulation of Bax and down regulation of Bcl2 during 3-NC mediated apoptosis in human cancer cells. Cancer Cell Int..

[42] Liu Y., Zuo H., Wang Y., Tian L., Xu X., Xiong J., Pei X. (2018). Ethanol promotes apoptosis in rat ovarian granulosa cells via the Bcl-2 family dependent intrinsic apoptotic pathway. Cell. Mol. Biol..

