# Supplementary material for: Hepatoprotective and Antioxidant Potential of Phenolics-Enriched Fraction of Anogeissus acuminata Leaf against Alcohol-Induced Hepatotoxicity in Rats
Source: Med Sci (Basel). 2022 Mar 4;10(1):17. doi: 10.3390/medsci10010017 (PMC8949889; doi:10.3390/medsci10010017)
Supplement: Supplementary file 1 [file medsci-10-00017-s001.zip › medsci-1554176-supplementary.pdf]

Table S1. The sequences of primers used in PCR reactions.

Gene	The sequence of primers (5′–3′)
**Bcl-2 F**	5'GTGGATGACTGAGTACCTGAAC 3'
**Bcl-2 R**	5' CAGCCAGGAGAAATCAAACAG 3'
**p53 F**	5'GCCATCTACAAGCAGTCACAG 3'
**p53 R**	5'TCATCCAAATACTCCACACGC 3'
**Bax F**	5'AGTAACATGGAGCTGCAGAG 3'
**Bax R**	5' AGTAGAAAAGGGCGACAACC 3'
**Caspase-3 F**	5'ACTGGACTGTGGCATTGAG 3'
**Caspase-3 R**	5'GAGCCATCCTTTGAATTTCGC 3'
**Caspase-9 F**	5' AGTTCCCGGGTGCTGTCTAT 3'
**Caspase-9 R**	5' GCCATGGTCTTTCTGCTCAC 3'
**GAPDH F**	5' GCATGGCCTTCCGTGTTCC 3'
**GAPDH R**	5' GGGTGGTCCAGGGTTTCTTACTC 3
